# Supplementary material for: Ready for handwriting? A reference data study on handwriting readiness assessments
Source: PLoS One. 2023 Mar 3;18(3):e0282497. doi: 10.1371/journal.pone.0282497 (PMC9983835; doi:10.1371/journal.pone.0282497)

PLOS ONE

Reference data for handwriting readiness assessment – using Writing Readiness Inventory In Context (WRITIC) and fine-motor coordination tests

--Manuscript Draft--

**Manuscript Number:**	PONE-D-22-29322
**Article Type:**	Research Article
**Full Title:**	Reference data for handwriting readiness assessment – using Writing Readiness Inventory In Context (WRITIC) and fine-motor coordination tests
**Short Title:**	Reference data for handwriting readiness assessment
**Corresponding Author:**	Liesbeth de Vries, MSc. OT<br>Amsterdam University of Applied Sciences<br>Amsterdam, NETHERLANDS
**Keywords:**	kindergarten; handwriting; schools; handfunction; early detection
**Abstract:**	Introduction: Early evaluation of writing readiness is essential to predict and prevent handwriting difficulties and its negative influences on school occupations. We developed an occupation-based measurement for kindergarten children: Writing Readiness Inventory Tool In Context (WRITIC). In addition, to assess fine motor coordination two tests are frequently used in children with handwriting difficulties: the Nine-Hole Peg Test (9-HPT) and the modified Timed Test of In-Hand Manipulation (Timed TIHM). However no Dutch reference data are available.<br>Aim: To provide reference data for (1) WRITIC, (2) Timed-TIHM and (3) 9-HPT for handwriting readiness assessment in kindergarten children.<br>Methods: Children in the age of 5 to 6.5 years were recruited at Dutch kindergartens. Full classes of the last year were tested. Descriptive statistics and percentiles scores were calculated. The score of the WRITIC and the performance time on the 9-HPT and Timed-TIHM are classified as percentile scores lower than the 15th percentile to distinguish low performance from adequate performance to identify children who are possibly at risk to develop handwriting difficulties in first grade.<br>Results: 374 children with a mean age of 5.6±0.4 years (190 boys, 184 girls) participated in the study. The mean WRITIC was 41 (SD 4.4), mean 9-HPT was 28.4 seconds (SD 5.4) and mean Timed-TIHM was 31.4 seconds (SD 7.4). A WRITIC score between 0-36, a performance time of more than 33.8 seconds on the 9-HPT and more than 39.6 seconds on the Timed-TIHM were classified as low performance.<br>Conclusion: The reference data of the WRITIC allow assessing which children are at risk to develop handwriting difficulties.
**Order of Authors:**	Helga Haberfehlner, PhD
	Liesbeth de Vries, MSc. OT
	Edith Cup, PhD
	Imelda de Groot, PhD
	Maria Nijhuis- van der Sanden, PhD
	Margo van Hartingsveldt, PhD
**Additional Information:**	
**Question**	**Response**
**Financial Disclosure**<br><br>Enter a financial disclosure statement that describes the sources of funding for the work included in this submission. Review the submission guidelines for detailed requirements. View published research	The author(s) received no specific funding for this work.

Powered by Editorial Manager® and ProduXion Manager® from Aries Systems Corporation

articles from *PLOS ONE* for specific examples.<br><br>This statement is required for submission and **will appear in the published article** if the submission is accepted. Please make sure it is accurate.<br><br>**Unfunded studies**<br>Enter: *The author(s) received no specific funding for this work.*<br><br>**Funded studies**<br>Enter a statement with the following details:<br>• Initials of the authors who received each award<br>• Grant numbers awarded to each author<br>• The full name of each funder<br>• URL of each funder website<br>• Did the sponsors or funders play any role in the study design, data collection and analysis, decision to publish, or preparation of the manuscript?<br>• **NO** - Include this sentence at the end of your statement: *The funders had no role in study design, data collection and analysis, decision to publish, or preparation of the manuscript.*<br>• **YES** - Specify the role(s) played.<br><br>* typeset	
**Competing Interests**<br><br>Use the instructions below to enter a competing interest statement for this submission. On behalf of all authors, disclose any competing interests that could be perceived to bias this work—acknowledging all financial support and any other relevant financial or non-financial competing interests.<br><br>This statement is required for submission and **will appear in the published article** if the submission is accepted. Please make sure it is accurate and that any funding sources listed in your Funding Information later in the submission form are also declared in your Financial Disclosure statement.	The authors have declared that no competing interests exist.

<table>
<tr><td>View published research articles from PLOS ONE for specific examples.

NO authors have competing interests

Enter: The authors have declared that no competing interests exist.

Authors with competing interests

Enter competing interest details beginning with this statement:

I have read the journal's policy and the authors of this manuscript have the following competing interests: [insert competing interests here]

* typeset</td><td></td></tr>
<tr><td>Ethics Statement

Enter an ethics statement for this submission. This statement is required if the study involved:

• Human participants
• Human specimens or tissue
• Vertebrate animals or cephalopods
• Vertebrate embryos or tissues
• Field research

Write "N/A" if the submission does not require an ethics statement.

General guidance is provided below. Consult the submission guidelines for detailed instructions. Make sure that all information entered here is included in the Methods section of the manuscript.</td><td>N/A</td></tr>
</table>

**Format for specific study types**<br><br>**Human Subject Research (involving human participants and/or tissue)**<br>• Give the name of the institutional review board or ethics committee that approved the study<br>• Include the approval number and/or a statement indicating approval of this research<br>• Indicate the form of consent obtained (written/oral) or the reason that consent was not obtained (e.g. the data were analyzed anonymously)<br><br>**Animal Research (involving vertebrate animals, embryos or tissues)**<br>• Provide the name of the Institutional Animal Care and Use Committee (IACUC) or other relevant ethics board that reviewed the study protocol, and indicate whether they approved this research or granted a formal waiver of ethical approval<br>• Include an approval number if one was obtained<br>• If the study involved *non-human primates*, add *additional details* about animal welfare and steps taken to ameliorate suffering<br>• If anesthesia, euthanasia, or any kind of animal sacrifice is part of the study, include briefly which substances and/or methods were applied<br><br>**Field Research**<br><br>Include the following details if this study involves the collection of plant, animal, or other materials from a natural setting:<br>• Field permit number<br>• Name of the institution or relevant body that granted permission	
**Data Availability**<br><br>Authors are required to make all data underlying the findings described fully available, without restriction, and from the time of publication. PLOS allows rare exceptions to address legal and ethical concerns. See the PLOS Data Policy and FAQ for detailed information.	Yes - all data are fully available without restriction

A Data Availability Statement describing where the data can be found is required at submission. Your answers to this question constitute the Data Availability Statement and **will be published in the article**, if accepted.<br><br>**Important:** Stating 'data available on request from the author' is not sufficient. If your data are only available upon request, select 'No' for the first question and explain your exceptional situation in the text box.<br><br>Do the authors confirm that all data underlying the findings described in their manuscript are fully available without restriction?	
**Describe where the data may be found in full sentences. If you are copying our sample text, replace any instances of XXX with the appropriate details.**<br><br>• If the data are **held or will be held in a public repository**, include URLs, accession numbers or DOIs. If this information will only be available after acceptance, indicate this by ticking the box below. For example: *All XXX files are available from the XXX database (accession number(s) XXX, XXX.).*<br>• If the data are all contained **within the manuscript and/or Supporting Information files**, enter the following: *All relevant data are within the manuscript and its Supporting Information files.*<br>• If neither of these applies but you are able to provide **details of access elsewhere**, with or without limitations, please do so. For example:<br><br>*Data cannot be shared publicly because of [XXX]. Data are available from the XXX Institutional Data Access / Ethics Committee (contact via XXX) for researchers who meet the criteria for access to confidential data.*<br><br>*The data underlying the results presented in the study are available from (include the name of the third party*	10.21943/auas.19236195

and contact information or URL).<br>• This text is appropriate if the data are owned by a third party and authors do not have permission to share the data.<br><br>* typeset	
Additional data availability information:	

Powered by Editorial Manager® and ProduXion Manager® from Aries Systems Corporation

Manuscript

Click here to access/download;Manuscript;Manuscript_Haberfehlner_et_al

Reference data for handwriting readiness assessment – using Writing Readiness Inventory In Context (WRITIC) and fine-motor coordination tests

Helga Haberfehlner[1], Liesbeth de Vries[2,3], Edith H. Cup,[4] Imelda J. de Groot[4], Maria W. Nijhuis-van der Sanden[5], and Margo J. van Hartingsveldt[2]

[1] Department of Rehabilitation Sciences, KU Leuven, Campus Bruges, Bruges, Belgium.

[2] Research Group Occupational Therapy, Urban Vitality, Centre of Expertise, Faculty of Health, Amsterdam University of Applied Sciences, Amsterdam, The Netherlands

[3] Occupational Therapy Programme, Hanze University of Applied Sciences, Groningen, The Netherlands

[4] Department of Rehabilitation, Donders Institute for Brain, Cognition and Behaviour, Radboud University Medical Center, Nijmegen, The Netherlands.

[5] Research Institute for Health Sciences, IQ healthcare, Radboud University Medical Center, Nijmegen, The Netherlands.

Correspondence should be addressed to Margo J. van Hartingsveldt;

m.j.van.hartingsveldt@hva.nl

Abstract

Introduction: Early evaluation of writing readiness is essential to predict and prevent handwriting difficulties and its negative influences on school occupations. We developed an occupation-based measurement for kindergarten children: Writing Readiness Inventory Tool In Context (WRITIC). In addition, to assess fine motor coordination two tests are frequently used in children with handwriting difficulties: the Nine-Hole Peg Test (9-HPT) and the modified Timed Test of In-Hand Manipulation (Timed TIHM). However no Dutch reference data are available.

Aim: To provide reference data for (1) WRITIC, (2) Timed-TIHM and (3) 9-HPT for handwriting readiness assessment in kindergarten children.

Methods: Children in the age of 5 to 6.5 years were recruited at Dutch kindergartens. Full classes of the last year were tested. Descriptive statistics and percentiles scores were calculated. The score of the WRITIC and the performance time on the 9-HPT and Timed-TIHM are classified as percentile scores lower than the 15th percentile to distinguish low performance from adequate performance to identify children who are possibly at risk to develop handwriting difficulties in first grade.

Results: 374 children with a mean age of 5.6±0.4 years (190 boys, 184 girls) participated in the study. The mean WRITIC was 41 (SD 4.4), mean 9-HPT was 28.4 seconds (SD 5.4) and mean Timed-TIHM was 31.4 seconds (SD 7.4). A WRITIC score between 0-36, a performance time of more than 33.8 seconds on the 9-HPT and more than 39.6 seconds on the Timed-TIHM were classified as low performance.

Conclusion: The reference data of the WRITIC allow assessing which children are at risk to develop handwriting difficulties.

Introduction

The time children spent on fine motor activities and handwriting in school has decreased over the last 20 years as technology has become more important in childhood education. However, primary school children still spend 18%-47% of classroom activities on fine motor activities mainly handwriting (1). Prewriting and handwriting therefore remain an important goal of education in primary school. In most western countries, children learn prewriting skills in kindergarten at an age of 5 to 6 years. In this phase, they learn an appropriate sitting posture, to handle the pencil properly and produce different writing patterns before they start handwriting with cursive or block letters. Learning to write is not easy for everyone, some children develop handwriting difficulties. The prevalence of handwriting difficulties in 6-12 old children ranges between 6% and 33% (2). Persistent handwriting difficulties may have negative effects on a child's academic performance and self-esteem (Ratzon, Efraim & Bart, 2007). Identification of kindergarten children at risk of developing handwriting difficulties may allow early intervention to prevent handwriting difficulties and negative secondary effects in later grades.

The Writing Readiness Inventory Tool In Context (WRITIC) is developed to evaluate handwriting readiness in 5-6.5 years old children (3). In previous studies, it has been found that the WRITIC is a valid, reliable, feasible and predictive measure (3-5). The WRITIC gives valuable criterion-referenced information on handwriting readiness by evaluating factors regarding the child, the environment, and the paper-and-pencil tasks (3). The subdomain 'Task performance' of the WRITIC (WRITIC-TP) will be the norm-referenced subdomain which clarifies if children are ready to learn the skill of handwriting (3). However, reference data are not yet available.

If children are not ready for handwriting, it has been suggested to assess performance components of handwriting such as fine motor coordination (4, 6, 7) and visual-motor integration (4, 8-10) since they have found to be prerequisites for handwriting (9). Visual-motor integration can be tested by the Beery-Buktenica Developmental Test of Visual-Motor Integration (Beery VMI) (11). For measuring fine motor coordination two tests have been found most feasible and are widely accessible (12): the Nine-Hole Peg Test (9-HPT) (13, 14) evaluates static fine motor skills and the Test of In-Hand Manipulation Revised (TIHM-R) (15), modified in the Timed Test of In-Hand Manipulation (Timed TIHM) (16) evaluates the dynamic fine motor skills of in-hand manipulation. For the Beery VMI applicable reference values are available (11), while for the 9-HPT only American reference values exist for the age group of 5-6.5 years (13, 14). For the Timed-TIHM no reference values are available.

The aim of the current study was to provide reference data for (1) WRITIC-TP, (2) Timed-TIHM and (3) 9-HPT for the assessment of handwriting readiness in kindergarten children (age 5-6.5 years). In addition, we assessed gender and age differences on WRITIC-TP, Timed-TIHM and 9-HPT and we evaluated the correlations between these three tests. Based on earlier research (3, 5, 16) our hypothesis is that both fine motor tests would have a moderate correlation with WRITIC-TP.

Materials and Methods

Participants

Children in the age of 5 to 6.5 years were recruited by asking 25 teachers of kindergarten classes of elementary schools for their participation. In the Netherlands children perform prewriting skills in kindergarten, before they start in grade one with un-joined cursive script

or block letters. Full classes were tested. Schools from different parts of the Netherlands were approached in order to represent the present cultural diversity of children in the Netherlands. Following personal characteristics of the child were registered: age rounded to full months, gender and dominate hand.

Ethical approval has been obtained from the Institutional Review Board of the Research Committee involving Human Subjects (Dutch abbreviation: CMO) of Radboudumc (Arnhem-Nijmegen region) under IRB no. 35498.091.11. Prior to participation, the parents of each child were informed of the study and asked to sign informed consent. Children were excluded if they were unable to complete the items of the WRITIC due to a medical diagnosis such as a visual, auditory, motor or intellectual impairment.

Instruments

Writing Readiness Inventory Tool In Context (WRITIC)

The WRITIC is an occupation-based assessment to evaluate handwriting readiness (3). The WRITIC contains items of three domains: 'Child', 'Environment', and 'Paper-and-pencil tasks'. Every domain consists of two subdomains: the 'Child' domain includes 'Interest' and 'Sustained attention', the 'Environment' domain includes 'Physical environment' and 'Social environment', and the Paper-and-pencil tasks domain includes 'Task performance' and 'Intensity of performance'. The WRITIC is administered in the classroom, where the influence of the context is taken into account. First, the child's interest in paper-and-pencil tasks is evaluated. Then, the child is encouraged to complete a drawing booklet with seven paper-and-pencil tasks while a trained assessor observes and scores performance and quality of the paper-and-pencil tasks.

The subdomain 'Task performance' (i.e. WRITIC-TP) within the 'Paper-and-pencil tasks' domain was developed as a norm-referenced part of the WRITIC. The WRITIC-TP consists of seven items scoring the quality of paper-and-pencil tasks (tracing double-lined paths, colouring, making arcades, making garlands, name writing, making spirals, and copying letters and numbers) on a 3-point scale (range 0-2, maximum score: 14), and the performance of these tasks regarding sitting posture and pencil grip: five items (sitting posture, forearm position, wrist position, distal versus proximal movement, and other hand) are scored on a 7-point scale with a range of 0 to 6 resulting in a maximum score of 30 and one item on a 5-point scale (range 0-4, maximum score: 4). So, the total score ranges from 0-48.

Previous research of the WRITIC-TP confirmed high internal consistency ($\alpha = 0.82$), significant ability to discriminate between children with good and poor performance on paper-and-pencil tasks ($U = 11.78$, $p < 0.001$), and excellent test-retest and inter-rater reliability, with ICC's of 0.92 and 0.95 respectively. The WRITIC-TP, administered in kindergarten, is found to be the main predictor for handwriting quality evaluated by the Systematic Screening for Handwriting Difficulties (17) in grade 1 (4). The WRITIC takes approximately 20 minutes to administer.

Timed Test of In-Hand Manipulation (Timed TIHM)

The Timed-TIHM assesses three skills of in hand manipulation: 1) translation from finger to palm; 2) translation from palm to finger; and 3) complex rotation of 360° (16). The test is designed for children from 5 to 6 years of age. It takes 5 to 7 minutes to administer. For the Timed-TIHM the commercially available 9-Hole Peg Test Kit is used. The child is asked to pick up successively two, three, four, and five pegs with the dominant hand, manipulate the pegs with the fingertips to the palm, and keep them in the palm of the hand (translation from finger

to palm with stabilization), and then to replace the pegs one by one into the pegboard (translation from palm to finger with stabilization). The tasks with two, three, four and five pegs are included as practice items, followed by two trials with five pegs to score the time. The third task is two trials with a complex rotation task in which the child is asked to rotate one peg 360^0 for a total of five times using the fingertips of the dominant hand. The best time score of the two trials is used as the outcome measure, with a low time score corresponding to good fine motor performance. The number of drops and the times on external surface that is used to compensate are recorded as supplemental qualitative information. The time of the two translation and two rotation tasks are added and used for further analysis.

Research with the previous version of this assessment confirmed inter-rater reliability and construct validity using Rasch modeling: the TIHM-Revised (15). Test-retest reliability of the Timed-TIHM was good with an ICC of 0.71. Convergent validity with the WRITIC-TP was moderate with $r_s = 0.40$ (16).

Nine-Hole Peg Test (9-HPT)

The 9-HPT evaluates simple fine motor patterns, including reaching, grasping, carrying, entering, and releasing with the time taken to perform these tasks as the outcome measure (13). The 9-Hole Peg Test Kit is a simple, commercially available timed test of fine motor coordination in which nine pegs are inserted one by one into a pegboard and then consecutively removed. In our research children were asked to complete the task twice with the dominant hand, which they used for paper-and-pencil tasks. The best time score was used, with a low score (less time needed to perform the task) corresponding to good fine motor performance.

The 9-HPT has been validated in an American study population of 826 children

between 5 and 10 years of age. High inter-rater and test–retest reliability was established, and strong construct validity was obtained. Normative values are available for children in this age category (13).

Procedure

Children were assessed on the WRITIC, the Timed-TIHM and the 9-HPT. The WRITIC was administered individually in the classroom during a time when all the children were doing different tasks in small groups. The Timed-TIHM and the 9-HPT were administered in the same session outside the classroom in a one-to-one situation.

Test administrators included 27 bachelor students of occupational therapy, exercise therapy and physical therapy. To become competent in administering the tests, all administrators 1) attended a full day training including theory of handwriting readiness and fine motor coordination, practicing scoring the WRITIC, the Timed-TIHM and the 9-HPT by means of videos of assessments and practicing administration of the three tests on each other; 2) practised WRITIC with two typically developing children; 3) assessed the first two children in the presence of another student in order to give each other feedback and guarantee consistence between the test administrators.

Statistical analysis

Raw data of WRITC-TP, Timed-TIHM and the 9-HPT were used for statistical analysis. Normal distribution was checked by means of a normal probability plot and tested by Kolmogorov-Smirnov test of normality. Children were divided in age groups of each 6 months (i.e.: 60-65 months = 5-.5.5 years; 66-71 months = 5.5-6 years; 72-78 months = 6-6.5 years). Scores of all three tests were compared among age groups and gender. Descriptive statistics and

percentiles scores were calculated. Non-parametric test (i.e. Mann-Whitney U Test to test for gender differences and Kruskal-Wallis Test to test for differences in age groups) were used, as data was not normally distributed.

Correlations between the three tests were calculated by Spearman's *rho* (ρ). 001). The criteria proposed by Hinkle and colleagues were used for interpretation: a correlation coefficient greater (lower) than 0.90 (-0.90) was considered very high positive (negative) correlation, 0.70 to 0.90 (-0.70 to -0.90) high positive (negative) correlation, 0.50 to 0.70 (-0.50 to -0.70) moderate positive (negative) correlation, 0.30 to 0.50 (-0.30 to -0.50) low positive (negative) correlation and 0.00 to 0.30 (-0.00 to -0.30) little if any correlation (18).

The group of children that scored on one of the three test lower than the 15th percentile were separately analyzed using a VENN-diagram to assess the overlap in scores on WRITIC-TP, 9-HPT and Timed-TIHM (19). Statistical analyses were performed using SPSS 22 (IBM SPSS Statistics, Amsterdam, The Netherlands). A p-value of less than.05 was considered to indicate a statistically significant difference.

Results

374 children with a mean age of 5.6 years (SD 0.4) (190 boys, 184 girls) participated in the study. 319 children were assessed on all three tests, while for 55 only the WRITIC was administered due to time constrains. 87% of the participants was right-handed, 12% left-handed and 1% variable-handed. In the younger age groups (5-5.5 years and 5.5-6 years) there were more children compared to the oldest group (6-6.5 years) (i.e. 42%, 44% and 14%, respectively). Demographics for the age groups are shown in Table 1.

Table 1: Descriptive data of participants tested by the WRITIC only (n=55) and by all three tests (WRITIC, 9-HPT and Timed-TIHM (n=319)**.**Temperature and wildlife count in the three areas covered by the study.

		Age category (Number (%))			Total group
		**5-5.5 years**	**5.5-6 years**	**6-6.5 years**	
**Number of participants**	**WRITIC only**	156	164	54	374
	*All three tests**	*154*	*138*	*27*	*319**
**Boys / girls**	**WRITIC only**	82 / 74 (52.6 % / 47.4%)	83 / 81 (50.6% / 49.4%)	25 / 29 (46.3% /53.7%)	190 / 184 (50.8% / 49.2%)
	*All three tests**	*81 / 73 (52.6% / 47.4%)*	*69 / 69 (50.0% /50.0%)*	*14 / 13 (51.4% /48.1%)*	*164 / 155 (51.4% / 48.6%)*
**Right / Left / variable-handed**	**WRITIC only**	134 /22/0 85.9% / 14.1%/ 0%)	145 / 18 / 1 (88.4% / 11.0% / 0.6%)	43 / 4 / 2 (87.8% / 8.2% / 4.1%)	327/ 44 / 3 (87.4% / 11.8% / 0.8 %)
	*All three tests**	*133 / 21 / 0 (86.4% / 13.6% / 0%)*	*120 / 17 / 1 (87.0% / 12.3% / 0.7 %)*	*24 / 2 / 1 (88.9% / 7.4% / 3.7%)*	*277 / 40 / 2 (86.8% /12.5%/0.6 %)*

WRITIC = Writing Readiness Inventory Tool In Context, 9-HTP = Nine-Hole Peg Test, Timed-TIHM = Timed Test of In-Hand Manipulation, % = percentage;

*Note that only 319 children were assessed on all three tests (cursive = subpopulation of total group, n=374.

The mean WRITIC-TP was 41.3 points (95% CI=40.8-41.7), not normally distributed (p<0.001, skewness of -1.05) with an SD of 4.4 points. Also 9-HPT (mean±SD: 28.4±5.4 seconds, 95% CI=27.8-29.0) and Timed-TIHM (mean±SD: 31.4±7.4 seconds, 95% CI=30.4-32.1) were not normally distributed but positively skewed (p<0.001, skewness of 0.91 and p<0.001, skewness=1.16, respectively) (Figure 1).

Figure 1: Frequency of points on (A) 'Task performance' of the Writing Readiness Inventory Tool In Context (WRITIC-TP), (B) seconds on Nine-Hole Peg Test (9-HPT) and (C) the Timed Test of In-Hand Manipulation(Timed-TIHM). WRITIC-TP is negatively skewed, 9-HPT and Timed-TIHM positively.

As data were not normally distributed, non-parametric statistics were applied. Note that for the Timed-TIHM the time value of all children that could perform the test is used. 4.1% of

children (n=13 out of 319 children) could not perform the rotation task of the Timed-TIHM and had therefore no time recorded on this test. The percentile scores of the Timed-TIHM were corrected for these 4.1% of children who were not able to perform the Timed-TIHM completely i.e. they were counted within the group with the lowest scores. The score of the WRITIC-TP and the performance time on the 9-HPT and Timed-TIHM that are classified as percentile scores lower than the 5th percentile, lower than the 15th percentile respectively, as well as the points and time that are classified higher than 15th percentile are displayed in table 2.

Table 2: Percentile scores of the subdomain 'Task performance' of the Writing Readiness Inventory Tool In Context (WRITIC-TP), the Nine-Hole Peg Test (9-HTP) and the Timed Test of In-Hand Manipulation (Timed-TIHM).

	**WRITIC-TP**	**9-HPT**	**Timed-TIHM**
**Percentile score**	**Raw score**	**Time**	**Time**
<5th percentile	0-32 points	>38.3 seconds	>56.0 seconds or not able to perform
5th-15th percentile	33-36 points	38.3-33.8 seconds	56.0-39.6-seconds
15th-50th percentile	37-41 points	33.9-27.3 seconds	39.7-30.1 seconds
50th – 85th percentile	42-44 points	27.4-23.2 seconds	30.2-24.5 seconds
85th -95th percentile	45-46 points	23.3-21.4 seconds	24.6-22.2 seconds
>95th percentile	47-48 points	<21.4 seconds	<22.2 seconds

Boys and girls performed slightly different on the WRITIC-TP with girls achieving more points on average on the WRITIC-TP (girls: 42.1±3.8 points; boys: 40.5±4.8 points; $p=0.001$). This difference between boys and girls on the WRITIC-TP was mainly determined by the difference in sum score of the items scoring the quality of results of the paper-and-pencil tasks ($p<0.001$), but not in the items scoring the quality of performance of these tasks (e.g. sitting position, pencil grip) ($p=0.732$). On the Timed-TIHM also a difference was found between boys and girls, with girls performing on average faster (girls: 30.3±6.9 seconds; boys: 32.4±7.7

seconds; p=0.006). No difference was found on the 9 HPT (p=0.281). For age groups we found no difference for the WRITIC-TP (5-.5.5 years: 40.9±4.4 points, 5.5-6 years: 41.6±4.6 points and 6-6.5 years: 41.6±3.9 points; p=0.239), but significant differences on the 9-HPT (5-5.5 years: 29.3±5.7 seconds, 5.5-6 years: 27.7±5.0 seconds and 6-6.5 years: 26.8±4.8 seconds) and Timed-TIHM (5-5.5 years: 32.6±8.1 seconds, 5.5-6 years: 30.1±6.5 seconds and 6-6.5 years: 30.4±6.4 seconds) (p=0.010 and p=0.032, respectively), with older children performing faster.

WRITIC-TP, 9-HPT and Timed-TIHM were all correlated: WRITIC-TP had a low negative correlation with the 9-HPT and Timed-TIHM (ρ= -0.285, p<0.001; ρ= -0.331, p<0.001, respectively). 9-HPT had a low positive correlation with the Timed-TIHM (ρ= 0.482, p<0.001).

The group of children that scored on one of the three tests lower than the 15th percentile consisted of 103 children (60 boys). A VENN-diagram in Figure 2 shows the overlap between these subgroups.

Figure 2: Group of children (n=103) that scored lower than the 15th percentile on the subdomain 'Task performance' of the Writing Readiness Inventory Tool In Context (WRITIC-TP), on the Nine-Hole Peg Test 9-HPT or the Timed Test of In-Hand Manipulation (Timed-TIHM). VENN-diagram shows the overlap of children between the three tests.

Out of the 46 children (30 boys) that scored lower than the 15th percentile on the WRITIC-TP, 21 children (16 boys) out of 46 (45.7 %), also had a time score on either the Timed-TIHM or the 9-HPT lower than the 15th percentile, while 8 children (4 boys) out of the 46 (17.4%) had a time lower than the 15th percentile on both other tests.

Discussion

With the current research we provide reference data for the WRITIC-TP, Timed-TIHM and 9-HPT for handwriting readiness assessment in kindergarten children (5 to 6.5 years old). Gender differences were found for the WRITIC-TP and the Timed-TIHM with girls performing better than boys, but not for the 9-HPT. No difference was found for age groups for the performance on the WRITIC-TP, but it was present for the fine motor coordination assessments (Timed-TIHM and 9-HPT). About half of the children that showed a low performance on the WRITIC-TP, also scored low on fine motor coordination assessed by Timed-TIHM and/or 9-HPT.

The moderate correlation between 9-HPT and Timed-TIHM (ρ= 0.482) in the current study is comparable to previous research (16). However, the correlation between the WRITIC-TP and the tests of fine motor performance (9-HPT and Timed-TIHM) was lower than previously reported, possibly due to a different composition of the population. In previous research the participants were selected by the teacher (two or three children with a good and two or three with a poor performance of paper-and pencil tasks according to the teacher) (16). In the current study we tested full classes.

As expected, we did not find a full overlap in children performing low on all tests as show in the VENN-diagram. This supports the theory that besides fine motor performance there are other performance components needed for handwriting readiness. We found in previous research that besides fine motor coordination also visual-motor integration, as measured with the Developmental Test for Visual-Motor Integration (Beery-VMI) (11), is an important performance component, and this was validated by other research (8-10). Besides fine motor coordination and visual-motor integration in our research on predictive value of

the WRITIC we also found sustained attention as a predictive factor of handwriting attainment (4) this was also validated by other research (20-22).

Although we found gender differences on the WRITIC-TP and Timed-TIHM, we decided not to provide separate reference values. This decision was based on the fact that children have to learn handwriting at a certain age, independent of their gender. We found gender differences for the items of the WRITIC-TP scoring the quality of paper- and pencil tasks, which need practice to achieve proficiency. There were no gender differences for the items scoring the quality of performance (e.g. sitting position, pencil grip). Nor did we find differences in gender on the 9-HPT. However, in contrast to the basic fine motor skill of the 9-HPT, the Timed-TIHM is a complex fine motor skill that needs practice, resulting in gender differences. This is line with the research on gender differences in motor learning of Dorfberger and colleagues (2009). The results of their study suggest that gender may be an important factor in motor performance and especially in motor learning. In this study the initial gap in handwriting speed in 9-year old children, in favour of the girls, may represent an advantage in prior experience; in this study boys, especially the older participants, may be able to close this gap, or even reverse it, when further practice takes place (23).

The reference values of the 9-HPT in the Dutch population are rather comparable with previous research in an American population, and shows a decrease in performance time with age (13, 14). We did not find differences in gender within the age range 5-6.5 year on the 9-HPT, while others found gender differences calculated within a wider age range (i.e. 4-18 years) and with a larger age groups (i.e. one year instead of a half year as used in the current study) (13, 14).

Our reference data are collected in Dutch kindergarten classes and are therefore limited to this population. In countries with a similar educational system, reference data

might be applicable. However, we recommend the collection of reference data within each country separately as education and daily routines may play a role and show cultural differences. Concerning assessed differences in age groups (i.e. we found that older children were performing faster on the 9-HPT and Timed-TIHM), the results are in the line what is expected. However, the results especially for the oldest group (i.e. 6-6.5 years old) should be interpreted with caution taking into account that less children were included in this group than in the younger age groups. The reason that less children are included in this group is that we measured children about six to nine months before children enter grade one (i.e. start learning to write), therefore we had relatively few children in the oldest group. We choose this period to assess handwriting readiness based on pragmatical reasons, because then there is still enough time for an intervention before the child actually starts learning handwriting (24). All reference data are presented for the whole age group (5-6.5); the unequal distribution within the age-groups is not relevant in using the reference data in assessing handwriting readiness.

A potential limitation of this study is the use of 27 different raters: there could be bias between them. However, in a former study we determined an excellent ICC of 0.95 ($p < 0.001$) inter-rater reliability of WRITIC-TP with students who got the same procedure on training in administering the WRITIC (5). Moreover, using different raters in this study fits the situation in practice. The students who collected the data attended a full day training on administering WRITIC, practised WRITIC with two typically developing children, and checked their first two administrations with a colleague. To become a reliable administrator, it is essential to follow this training.

Conclusions

The reference data of the WRITIC-TP show which children are at risk to develop handwriting difficulties. When children have minor difficulties according to the WRITIC-TP, kindergarten teachers can be advised and supported in training the children in the mastery of prewriting skills to prevent handwriting difficulties in higher grades. When children have more complex difficulties, school-based occupational therapy is indicated whereby the occupational therapist works collaboratively with the teacher and parents. When children score below the 15th percentile and they are not ready for handwriting based on the WRITIC, additional assessments of performance components (fine motor coordination and visual-motor integration) can be used as second step in the evaluation of handwriting readiness. Based on this two-step assessment interventions to support the child's participation include adaptations in the child's physical and social environment such as a pencil grip, a different place in the classroom or an adapted instruction, task-oriented training with enough practicing time and demonstrating strategies that enhance participation of children in paper-and-pencil tasks (25-27).

Acknowledgments

The authors wish to thank the students who administered the tests in the children: Laudia Borsch, Jill de Haan, Anna Hildebrand, Kelly Huigsloot, Danielle Meijer, Lianne Stark, Michelle van Damme, Carina Dubbeldam, Naära Tomasowa (former students of the bachelor 'Occupational therapy' of Amsterdam University of Applied Sciences) and Aline Averesch, Iris Steijn, Jessica Spaan, Lisan Brookhuis, Lisette Weijers, Michiel van Lingen, Myrthe Verhoog, Mitchell Walgien, Ronalys de Gier, Siem Matton and Tom Smits (former

students of the Minor program ‘Child’ of the faculty of Health of Amsterdam University of Applied Sciences) and Deborah Cabal, Marieke Hagemeijer, Karin Stellingwerf (former students of bachelor ‘Exercise therapy’ of Amsterdam University of Applied Sciences) and Gerben ter Riet for his advice on the statistical analysis of the data.

References

1. McMaster E, Roberts T. Handwriting in 2015: A main occupation for primary school–aged children in the classroom? Journal of Occupational Therapy, Schools, & Early Intervention. 2016;9(1):38-50.

2. Overvelde A, Hulstijn W. Handwriting development in grade 2 and grade 3 primary school children with normal, at risk, or dysgraphic characteristics. Res Dev Disabil. 2011;32(2):540-8.

3. van Hartingsveldt MJ, de Vries L, Cup EH, de Groot IJ, Nijhuis-van der Sanden MW. Development of the writing readiness inventory tool in context (WRITIC). Phys Occup Ther Pediatr. 2014;34(4):443-56.

4. van Hartingsveldt MJ, Cup EH, Hendriks JC, de Vries L, de Groot IJ, Nijhuis-van der Sanden MW. Predictive validity of kindergarten assessments on handwriting readiness. Res Dev Disabil. 2015;36C:114-24.

5. van Hartingsveldt MJ, Cup EH, de Groot IJ, Nijhuis-van der Sanden MW. Writing Readiness Inventory Tool in Context (WRITIC): reliability and convergent validity. Aust Occup Ther J. 2014;61(2):102-9.

6. Berninger VW, May MO. Evidence-based diagnosis and treatment for specific learning disabilities involving impairments in written and/or oral language. J Learn Disabil. 2011;44(2):167-83.

7. Feder KP, Majnemer A, Bourbonnais D, Platt R, Blayney M, Synnes A. Handwriting performance in preterm children compared with term peers at age 6 to 7 years. Dev Med Child Neurol. 2005;47(3):163-70.

8. Daly CJ, Kelley GT, Krauss A. Relationship between visual-motor integration and handwriting skills of children in kindergarten: a modified replication study. Am J Occup Ther. 2003;57(4):459-62.

9. Volman MJ, van Schendel BM, Jongmans MJ. Handwriting difficulties in primary school children: a search for underlying mechanisms. Am J Occup Ther. 2006;60(4):451-60.

10. Marr D, Windsor MM, Cermak S. Handwriting Readiness: Locatives and Visuomotor Skills in the Kindergarten Year. Early Childhood Research & Practice. 2001;3(1).

11. Beery K, Buktenica NA, Beery NA. Beery-Buktenica Developmental Test of Visual Motor Integration (Beery VMI). 6th ed. San Antonio: Pearson; 2010.

12. van Hartingsveldt MJ, De Groot IJ, Aarts PB, Nijhuis-Van Der Sanden MW. Standardized tests of handwriting readiness: a systematic review of the literature. Dev Med Child Neurol. 2011;53(6):506-15.

13. Smith YA, Hong E, Presson C. Normative and validation studies of the Nine-hole Peg Test with children. Percept Mot Skills. 2000;90(3 Pt 1):823-43.

14. Poole JL, Burtner PA, Torres TA, McMullen CK, Markham A, Marcum ML, et al. Measuring dexterity in children using the Nine-hole Peg Test. J Hand Ther. 2005;18(3):348-51.

15. Pont K, Wallen M, Bundy A, Case-Smith J. Reliability and validity of the Test of In-Hand Manipulation in children ages 5 to 6 years. Am J Occup Ther. 2008;62(4):384-92.

16. de Vries L, van Hartingsveldt MJ, Cup EH, Nijhuis-van der Sanden MW, de Groot IJ. Evaluating fine motor coordination in children who are not ready for handwriting: which test should we take? Occup Ther Int. 2015;22(2):61-70.

17. Smits-Engelsman BC, Stevens M, Vrenken I, van Hagen A. Systematische Opsporing Schrijfproblemen(SOS): een hulpmiddel voor leerkrachten bij het signaleren van motorische schrijfproblemen van leerlingen in het Basis en Speciaal Onderwijs. [Systematic screen-ing of handwriting problems (SOS): an instrument for teachers for screening of handwriting problem of children in primary school and special education]. : . Kinderfysiotherapie. 2005;December (17):16–20.

18. Hinkle DE, Wiersma W, Jurs SG. Applied statistics for the behavioral sciences. 5th ed. ed. Boston, Mass., London: Houghton Mifflin ; Hi Marketing (distributor); 2003.

19. Micallef L, Rodgers P. eulerAPE: drawing area-proportional 3-Venn diagrams using ellipses. PLoS One. 2014;9(7):e101717.

20. Tseng MH, Chow SM. Perceptual-motor function of school-age children with slow handwriting speed. Am J Occup Ther. 2000;54(1):83-8.

21. Grissmer D, Grimm KJ, Aiyer SM, Murrah WM, Steele JS. Fine motor skills and early comprehension of the world: two new school readiness indicators. Dev Psychol. 2010;46(5):1008-17.

22. Cameron CE, Brock LL, Murrah WM, Bell LH, Worzalla SL, Grissmer D, et al. Fine motor skills and executive function both contribute to kindergarten achievement. Child Dev. 2012;83(4):1229-44.

23. Dorfberger S, Adi-Japha E, Karni A. Sex differences in motor performance and motor learning in children and adolescents: an increasing male advantage in motor learning and consolidation phase gains. Behav Brain Res. 2009;198(1):165-71.

24. Zylstra SE, Pfeiffer B. Effectiveness of a Handwriting Intervention With At-Risk Kindergarteners. Am J Occup Ther. 2016;70(3):7003220020p1-8.

25. Overvelde A, Smits-Engelsman BC, Nijhuis-Van Der Sanden MW. Motorische schrijfproblemen. In: Empelen R, Nijhuis-Van Der Sanden MW, Hartman A, editors. Kinderfysiotherapie. Amsterdam: Reed Business Education; 2013. p. 771-94.

26. Missiuna CA, Pollock NA, Levac DE, Campbell WN, Whalen SD, Bennett SM, et al. Partnering for change: an innovative school-based occupational therapy service delivery model for children with developmental coordination disorder. Can J Occup Ther. 2012;79(1):41-50.

27. Hoy MM, Egan MY, Feder KP. A systematic review of interventions to improve handwriting. Can J Occup Ther. 2011;78(1):13-25.

Figure 1

Click here to access/download;Figure;figure 1.jpg

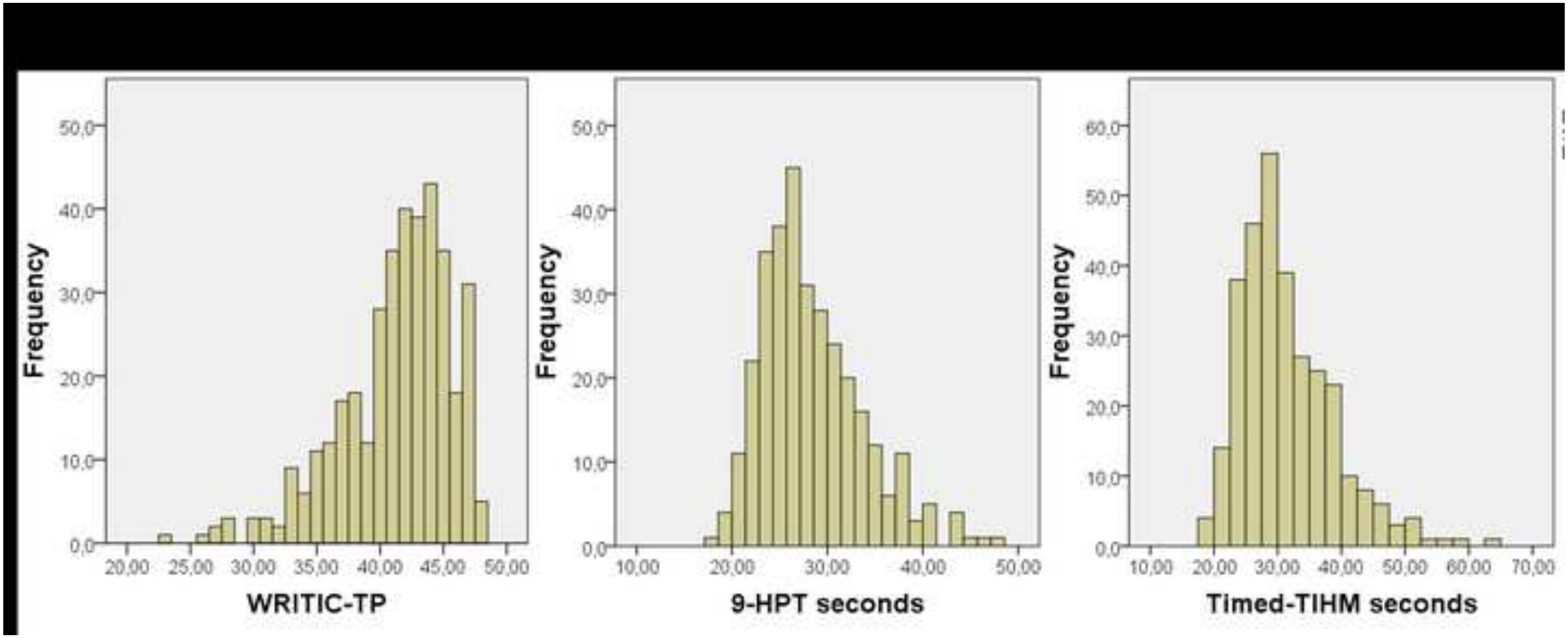

Figure 2

Click here to access/download;Figure;figure 2.jpg

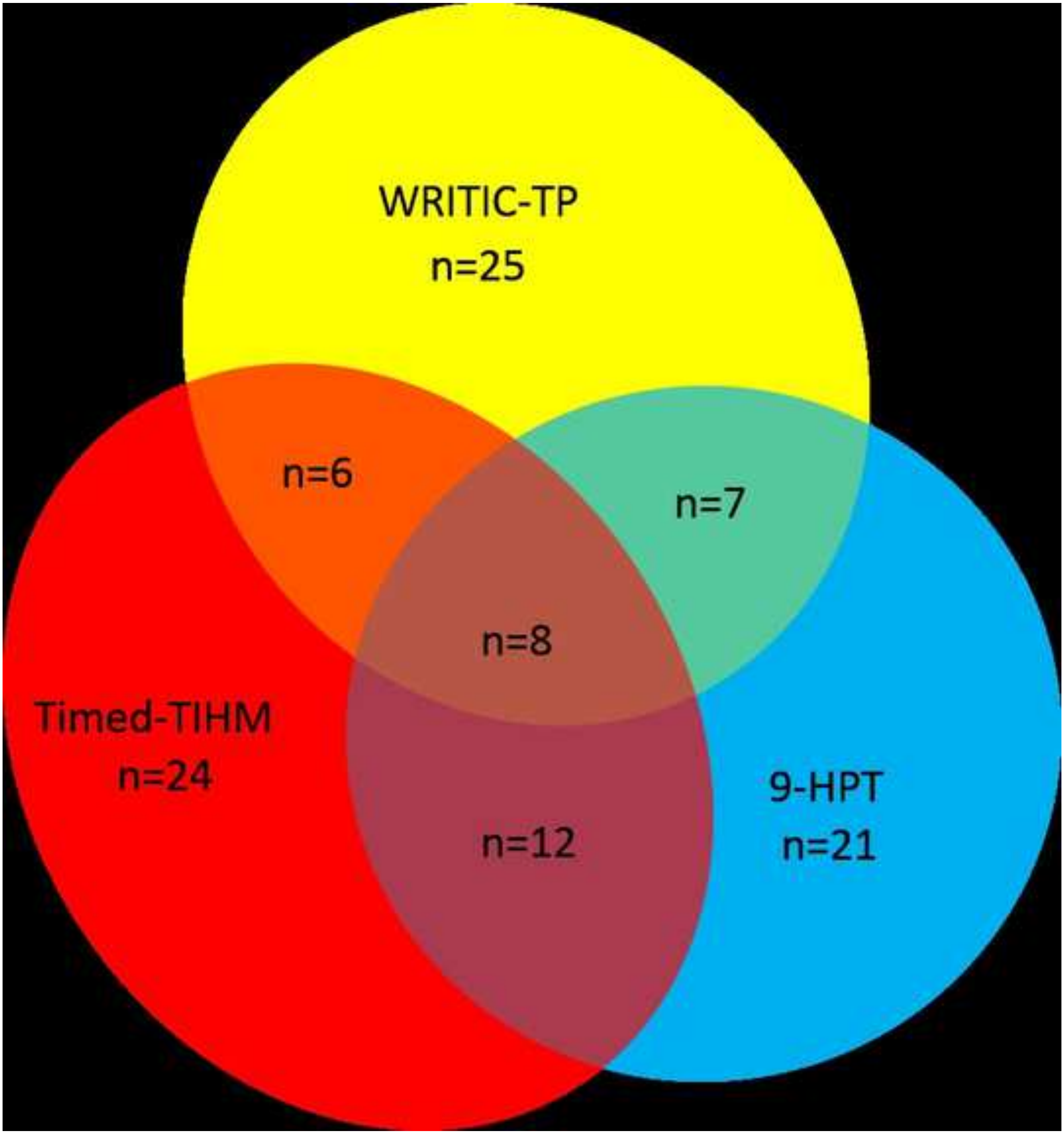

Supplement: Attachment — Submitted filename: PONE-D-22-29322- Revised.pdf [file pone.0282497.s002.pdf]
