# Supplementary material for: Ready for handwriting? A reference data study on handwriting readiness assessments
Source: PLoS One. 2023 Mar 3;18(3):e0282497. doi: 10.1371/journal.pone.0282497 (PMC9983835; doi:10.1371/journal.pone.0282497)
Supplement: Attachment — Submitted filename: Reference data for handwriting readiness assessment.docx [file pone.0282497.s001.docx]

Reference data for handwriting readiness assessment – using Writing Readiness

Inventory In Context (WRITIC) and fine-motor coordination tests

PONE-D-22-29322

Reviewer Becher

The manuscripts describes the results of the WRITEC in a typical developing population children, mean age 5,6 yrs (SD 0,4 yrs) of a Dutch population. To get data about the validity, the nine-hole peg test and the Timed Test in Hand Manipulation were scored.

Percentile scores were calculated, children with a score below the 15^th^ percentile were supposed to be at risk for developing writing problems at the start of learning writing.

Abstract

The abstract is clearly written.

P8 S 40

The concusion “Conclusion: The reference data of the WRITIC allow assessing which children are at risk to develop handwriting difficulties.” Does not fully fit the contents: low performance is described, but the relation with writing problems later in the development has not been demonstrated: the are possibly at risk.

P 9 s58

“in previous studies …. Etc” In these studies, only statistical analysis has been performed for groups. `the results from group studies is translated to individual risk. That is not valid: other statistics are used to predict the chance for an individual to get problems with learning writing, with a confidence interval. So, I propose to add “ group studies”.

P10 S 67-75

The tests described are all “laboratory” tests (capacity tests) of a specific construct, supposed to be important to learn writing. However, for all these tests it is not demonstrated on individual level to what extend they really predict problems with learning writing, with specificity and sensitivity. I proposed to add a remark that these capacities are supposed to have a relation with the development of writing problems, but the predictive value is unknown.

The participants and methods section is clearly described.

P 12 S 118: Cronbach’s alpha?

Results are properly described.

About the characteristics of the study population, nothing is mentioned about the background of the children. In the Netherlands, a lot of non-native families are present, in big cities up to 60% speak at home other languages than Dutch. There are cultural differences in raising up children, participation in kindergarten and playing at home.

Are the reference values mainly based on results of children of white Dutch families?

Discussion

P19 S 260

“No difference was found for age groups for the 261 performance on the WRITIC-TP,”

The authors do not comment on this remarkable findurthering: if the WRITIC-TP has a predictive value for learning writing, you expect higher scores with age. How can the authors explain this finding?

Conclusion

P22 S 326

Children are possibly at risk for

P22 S327-9

“When children have minor difficulties according to the WRITIC-TP, kindergarten teachers can be advised and supported in training the children in the mastery of prewriting skills to prevent handwriting difficulties in higher grades.”

The study does not provide any evidence for this statement and should be skipped.

Further research is needed to investigate the predictive value of a low WRITIC-TP score for development of writing problems at school.

The remark that occupational therapy is indicated is also beyond the contents of the research, please stick to the results of your results and remove personal statements.
