# Supplementary material for: Ready for handwriting? A reference data study on handwriting readiness assessments
Source: PLoS One. 2023 Mar 3;18(3):e0282497. doi: 10.1371/journal.pone.0282497 (PMC9983835; doi:10.1371/journal.pone.0282497)
Supplement: Attachment — Submitted filename: Response to reviewers.docx [file pone.0282497.s003.docx]

Response to reviewers PONE-D-22-29322

We would like to thank the editor and the reviewers for their very constructive and positive feedback on our manuscript. Below, we give a point-by-point response to the various comments and describe how we have addressed these in our revised manuscript. The original remarks are printed in normal font, our response is given in *italics.* Changed parts of the original text are quoted and identified using ‘track changes’ in the revised manuscript. Lines numbers refers to the “Revised manuscript with Tracked Changes”.

Comments editor:

1) I think the Title could be a little bit more intuitive. I have no concern regarding the way it is, but you can a more "punchy" title to gather other's attention to it;

*Reply:* *Thank you for your suggestion for a more “punchy” title, we change the title to*

“Ready for handwriting? A reference data study on handwriting readiness assessments”

(page 1: lines 3-4).

2) In abstract, please check some important issues: (i) provide mean age and SD for your sample in Methods instead of Results; (ii) do not begin the sentence with numbers, the authors need to change "Results: 374" to "Results: Three hundred and seventy four" and this needs to be constant thorough the text; and (iii) the WRITIC scores range (from x to x, for example);

*Reply: Thank you for your comment. The abstract was changed accordingly. In addition numbers in the beginning of the sentence are now written full out though the text. (page 1: lines 30-42; page 11: line: 217)*

3) Your eligibility criteria need to be better emphasised;

*Reply: Egiglibitly criteria were added to the abstract: “*Full classes of the last year were tested, children were excluded if there was a medical diagnosis such as a visual, auditory, motor or intellectual impairment that hinder handwriting performance.” *(page 1: line 32-34)*

4) Based on the skewed sample, this reminds me of the presentation of kurtosis and (why not?) conduct log-transform or fractional rank?

*Reply: We have now presented the kurtosis in the result section for the* WRITIC-TP, 9-HPT and Timed-TIHM *(page 12: lines 229-233). We chose to use raw data to calculate the percentile scores for the reference data without a transformation, as the raw data are most accessible for clinicians. The percentile scores account for the non-normal distribution with larger ranges for low percentiles and smaller ranges for high percentiles.*

5) Please clarity why you only provided descriptive of WRITIC and nothing on exploratory factor analysis? Was WRITIC previously validated in the same sample? If yes, what is the novelty of the study? If no, why can't the authors provide EFA as a sup. file for this specific sample?

*Reply: Thank you for your comment.* *In the WRITIC development process, we determined two subdomains of the Paper-and-Pencil Tasks domain based on factor analysis: Task Performance and Intensity of Performance. Task Performance (TP) is the norm-referenced subdomain of WRITIC, contains 13 items and had high internal consistency (α = 0.82). Analysis were performed during the development within a different sample (Ref 8 in the manuscript: van Hartingsveldt MJ, de Vries L, Cup EH, de Groot IJ, Nijhuis-van der Sanden MW. Development of the writing readiness inventory tool in context (WRITIC). Phys Occup Ther Pediatr. 2014;34(4):443-56.). The aim for current study was to present reference data for the WRITIC-TP within a new sample.*

6) Please avoid the use of paragraphs without proper references in Discussion. And check outdated references to ensure transparency and an updated manuscript.

*Reply: Thank you for your comment, we have updated references in the discussion.*

- *Page 5; lines 84 and Page 16, line 305 – we have added updated references that validate visual-motor integration as an important performance component.*
  - *14.	Dere Z. Analyzing the Early Literacy Skills and Visual Motor Integration Levels of Kindergarten Students. Journal of Education and Learning. 2019;8(2):176-81.*
  - *25.	Pinto G, Incognito O. The relationship between emergent drawing, emergent writing, and visual‐motor integration in preschool children. Infant and Child Development. 2022;31(2):e2284.*
- *Page 17 line 322– we added a reference updating the gender effect in improving fine motor skills in kindergarten and first grade children*
  - *25. Taverna L, Tremolada M, Dozza L, Zanin Scaratti R, Ulrike D, Lallo C, et al. Who benefits from an intervention program on foundational skills for handwriting addressed to kindergarten children and first graders? International Journal of Environmental Research and Public Health. 2020;17(6):2166.*
- *Page 17 line 334 – we have added a reference that contributes to the validation of the 9-HPT reference data.*
  - *30.	Omar MT, Alghadir AH, Zafar H, Al Baker S. Hand grip strength and dexterity function in children aged 6-12 years: A cross-sectional study. J Hand Ther. 2018;31(1):93-101.*
- *Page 18, line 363 – we have updated references regarding effective pre-writing interventions in kindergarten children.*
  - *32.	Kadar M, Chai SC, Geoh MK, Razaob NA, Yunus FW. Review on the Effect of Occupational Therapy Intervention in Improving Handwriting Skills among Preschool Children. Jurnal Sains Kesihatan Malaysia (Malaysian Journal of Health Sciences). 2019;17(2).*
  - *33.	Kadar M, Wan Yunus F, Tan E, Chai SC, Razaob@ Razab NA, Mohamat Kasim DH. A systematic review of occupational therapy intervention for handwriting skills in 4–6 year old children. Australian Occupational Therapy Journal. 2019.*

7) Finally, please double check English and the refs. list accordingly to the Journal's standard.

*Reply: We have double check English and corrected when necessary. We have also checked the reference list.*

Additional requirements:

1. Please ensure that your manuscript meets PLOS ONE's style requirements, including those for file naming. The PLOS ONE style templates can be found at

<https://journals.plos.org/plosone/s/file?id=wjVg/PLOSOne_formatting_sample_main_body.pdf>

and

<https://journals.plos.org/plosone/s/file?id=ba62/PLOSOne_formatting_sample_title_authors_affiliations.pdf>

*Reply: Thank you. Done.*

2. In your Data Availability statement, you have not specified where the minimal data set underlying the results described in your manuscript can be found.

*Reply: De data supporting our manuscript can be found on Figshare.* <https://doi.org/10.21943/auas.19236195.v1>.*We have added a data availably statement in the manuscript (page 19: lines 392-394)*

Comments Reviewer #1:

1) The abstract is clearly written.
   *Reply: Thank you*
2) P8 S 40
   The conclusion “Conclusion: The reference data of the WRITIC allow assessing which children are at risk to develop handwriting difficulties.” Does not fully fit the contents: low performance is described, but the relation with writing problems later in the development has not been demonstrated: they are possibly at risk.

*Reply: Thank you for your comment, we added the word ‘possibly’ on page 3, line 46 and on page 19, line 374. In addition, we added the reference of our study that underpins the predictive validity of the WRITIC (Page 8: line:145).*

3) P 9 s58
   “in previous studies …. Etc” In these studies, only statistical analysis has been performed for groups. `the results from group studies is translated to individual risk. That is not valid: other statistics are used to predict the chance for an individual to get problems with learning writing, with a confidence interval. So, I propose to add “group studies”.

*Reply: Thank you for your comment, we added the word ‘group’ (Page 5: line 75)*

4) P10 S 67-75
   The tests described are all “laboratory” tests (capacity tests) of a specific construct, supposed to be important to learn writing. However, for all these tests it is not demonstrated on individual level to what extend they really predict problems with learning writing, with specificity and sensitivity. I proposed to add a remark that these capacities are supposed to have a relation with the development of writing problems, but the predictive value is unknown.

*Reply: Thank you for your comment. In the psychometric studies we conducted on the development of WRITIC, we also conducted a predictive validity study (n=109) and found that WRITIC-TP and 9-HPT are both predictive of handwriting achievement, measured with the Systematic Screening for Handwriting problems (Dutch abbreviation: SOS)*

5) P 12 S 118: Cronbach’s alpha?

*Reply: Yes, we wrote it full out now (Page 8: lines 140-141)*

6) Results are properly described.
   About the characteristics of the study population, nothing is mentioned about the background of the children. In the Netherlands, a lot of non-native families are present, in big cities up to 60% speak at home other languages than Dutch. There are cultural differences in raising up children, participation in kindergarten and playing at home.
   Are the reference values mainly based on results of children of white Dutch families?

*Reply: Thank you for the comment. We indeed only stated: “*Schools from different parts of the Netherlands were approached in order to represent the present cultural diversity of children in the Netherlands.” (Page 7: lines 107-109). *However, we registered nonwestern migration background of children to control for the distribution and had 6-16% children with migration background in our group, which is in line with numbers released by Statistics Netherlands (CBS) for the Netherlands. We added the information about the background in* Table 1: Descriptive data of participants *(page: 11: line 225-227). The percentage of children with a migration background is highest in the oldest group (6-6.5 years). This can be explained by the fact that in the Netherlands there is no strict age for entering first grade. Children with a delay often attend kindergarten for an extra year in order to be optimally prepared. The standard age to enter first grade in the Netherlands (school year starts at the end of August) is 5.9 years (born till end of December) – 6.9 years (born from the beginning of January). We included children six to nine month before entering grade one, so within the standard age range from first grade one, this would be 5 years – 6.3 years. However, we did not exclude children who duplicated one year of kindergarten, but still used a cut off of 6.5 years.*

Discussion

7) P19 S 260
   “No difference was found for age groups for the 261 performance on the WRITIC-TP,”
   The authors do not comment on this remarkable finding: if the WRITIC-TP has a predictive value for learning writing, you expect higher scores with age. How can the authors explain this finding?

*Reply: This finding is most likely related to our comment on your previous question. Only children are tested who will start grade one within the next six to nine months, thus this children are expected to be similarly prepared concerning their prewriting skills. In addition, in the older group also children are included with some developmental delays, which made them to duplicate the last kindergarten year. We added a comment to this within the discussion. It reads as follow:* “In addition, some of the older children might have duplicated the last kindergarten year due to developmental delays and therefore show lower performance on the WRITIC-TP.” (Page 17: lines 346-348)

Conclusion
P22 S 326
Children are possibly at risk for
 *Reply: “possibly” is added to the conclusion. (Page 19: line: 374). Thank you.*

8) P22 S327-9
   “When children have minor difficulties according to the WRITIC-TP, kindergarten teachers can be advised and supported in training the children in the mastery of prewriting skills to prevent handwriting difficulties in higher grades.”
   The study does not provide any evidence for this statement and should be skipped.
   Further research is needed to investigate the predictive value of a low WRITIC-TP score for development of writing problems at school.
   The remark that occupational therapy is indicated is also beyond the contents of the research, please stick to the results of your results and remove personal statements.

*Reply: We agree with this comment. We skipped this information from the conclusion.*

Reviewer #2

1) The work presents important results for interventions and the creation of strategies that assist in child development, mainly ensuring the standardization of measures that can be used mainly as screening in the school context. I would like to congratulate the authors because this type of work is extremely important and aids in child development through a multidisciplinary dialogue.
   In general, the work presents current references (following the APA standard), objective language, and robustness of data analysis. The following highlights some suggestions for possible changes aimed at the authors.

*Reply: We thank the reviewer for the compliments on our work.*

Introduction

2) This section is very objective and presents relevant topics for understanding the importance of writing learning processes.
   However, I recommend adding on the benefits that arise from these processes and deepening how difficulties in this sense can influence development globally.

*Reply: We agree with this comment and have added the benefits of handwriting for the learning of reading and spelling. This reads as follows:* “Despite the increasing use of computers, tablets, and smartphones, handwriting remains an important skill that children learn to participate in school (1). Handwriting is essential for learning reading and spelling. The brain has close functional relationships between the reading and writing processes(2). James and Engelhart stated that handwriting is important for the early conscription in letter processing of brain regions known to underlie successful reading and may facilitate reading achievement in young children (3).” *(Page 4: lines 49-54)*

3) I also recommend deepening the "negative side effects in later series" cited on page 3 of the manuscript.

*Reply: Thank you for your suggestion, we have delved into the negative effects of handwriting problems a bit more and adapted this part of the introduction: “*It has been reported that children with handwriting difficulties develop negative experiences in this area, including frustration, self-reliance, and low motivation(6). Persistent handwriting difficulties may also have negative effects on a child’s academic performance and self-esteem (7). ” (Page 4: lines 68-71)

4) In the introduction, it is important that the reader can glimpse possible connections between manual writing and integration with other aspects evaluated, such as visuomotor skills. Thus, I recommend the addition of a paragraph that presents some studies that have evidenced these relationships and the importance of developing these pre-handwriting skills.

*Reply: We completely agree that it is important to give some insights on possible connections between fine motor coordination and visual-motor integration. This was already mentioned in our manuscript briefly: “*If children are not ready for handwriting, it has been suggested assessing performance components of handwriting. This assessment should include fine motor coordination (9, 11, 12) and visual-motor integration (6, 9, 13, 14) since these components have been found to be prerequisites for handwriting (13). Visual-motor integration can be tested by the Beery-Buktenica Developmental Test of Visual-Motor Integration (Beery VMI) (15). For measuring fine motor coordination two tests have been found most feasible. The first is the Nine-Hole Peg Test (9-HPT) (16, 17) which evaluates static fine motor skills and is widely accessible (18). The second is the Test of In-Hand Manipulation Revised (TIHM-R) (19).” (page 5 : lines 83-90)

Materials and methods

5) In the text, the ethical aspects of research and data collection are adequately presented.
   However, I recommend deepening in relation to the exclusion criteria used.
   In the abstract, it is stated that no data had previously been collected using the instruments chosen with a Dutch sample.
   However, it is unclear whether such instruments had previously been adapted for this population. In order to avoid possible misunderstandings regarding the application and use of the instruments, I recommend adding a brief sentence quoting the versions used in the work and clarifying these previously highlighted points.

*Reply: Thank you for your comment and we can imagine that it is not entirely clear to the reader which version of the fine motor coordination tests we used in our research. At the end of the introduction, we now clarified the information about the fine motor coordination tests (Page 5: line 89-92) and also in the section Method - Instruments we mentioned that the validity of this version was determined in a Dutch population (Page 9: line 165-167 )*

Procedure

6) In the text, it is highlighted that the "Timed-TIHM and 9-HPT were administered in the same session outside the classroom in an individual situation." Since the instruments were applied in the same session, I recommend adding more details about how the session was handled—could the child request to stop during the application? Was fatigue considered a variable that could influence?

*Reply: Thank you for your comment. We have added some details about fatigue and random order of the fine motor skills tests and about the ability to stop when children indicate this. This read as follows: “* The Timed-TIHM and the 9-HPT were administered in the same session outside the classroom in a one-to-one situation, in a random order due to possible fatigue. According to the informed consent, children could stop if they indicated*.” (Page 10: line 186-187)*

Statistical analysis

7) The authors presented in an appropriate and explanatory way the analyzes used, in this section, I only recommend the addition of a reference highlighted in the manuscript.

*Reply: Unfortunately we do not understand your recommendation as we have posted Hinkle and colleagues' reference on the interpretation of the Spearman's rho and Micallef and colleagues' reference on using a Venn diagram. All statistical test were performed in SPSS 22.*

Results

8) The findings were presented adequately, both in text format and in the table.

*Reply:* *We thank the reviewer for these compliments*

Discussion

9) The importance of considering cultural and teaching differences from other countries in future applications and study replications is addressed. I believe it is critical to emphasize the characteristics of Dutch teaching that the authors believe differ from other studies and how this influences the results obtained.

*Reply: Thank you for your comment. In the introduction we added information about the characteristics of the Dutch school system with regard to learning handwriting. This reads as follows: “*. In the Netherlands children learn prewriting skills in kindergarten at an age of 5 to 6 years. In this phase, children learn an appropriate sitting posture, to handle the pencil properly and produce different writing patterns before they start handwriting with cursive or block letters in first grade. In the Netherlands, children enter first grade normally in the school year in which they turn six before 1 January. However, teachers can decide together with parents to let children duplicated the last kindergarten year due to developmental or social delays.” (page 4: lines 63-66)

I recommend including a higher resolution version of image 1 in particular. Regarding image 2, I recommend changing the flowchart format or transposing the data to a table.
*Reply: We have changed the Figures to a higher resolution. Thank you for pointing out. Concerning Figure 2: We find the overlap and not-overlap quite informative. If the reviewer still recommend to change it, we are willing to transform the figure.*
