# Supplementary material for: Ready for handwriting? A reference data study on handwriting readiness assessments
Source: PLoS One. 2023 Mar 3;18(3):e0282497. doi: 10.1371/journal.pone.0282497 (PMC9983835; doi:10.1371/journal.pone.0282497)
Supplement: Attachment — Submitted filename: Reference data for handwriting readiness assessment V2.docx [file pone.0282497.s004.docx]

Reference data for handwriting readiness assessment – using Writing Readiness

Inventory In Context (WRITIC) and fine-motor coordination tests

PONE-D-22-29322R1

Reviewer Becher

The authors improved the manuscript considerable according the remarks of the reviewers.

I am satisfied with the comments on my remarks from the first review.

I have 1 remark about the change of the introduction:

Introduction

P 35 S 50

Ref 3: in summary: “Preliterate, five-year old children printed, typed, or traced letters and shapes, then were shown images of these stimuli while undergoing functional MRI scanning. A previously documented "reading circuit" was recruited during letter perception only after handwriting-not after typing or tracing experience. These findings demonstrate that handwriting is important for the early recruitment in letter processing of brain regions known to underlie successful reading. Handwriting therefore may facilitate reading acquisition in young children.”

The reading circuit was only activated by handwriting in healthy children.

However, there are dyslexic children who can read at normal level but are of very low level in writing / spelling. Children with weakness of the arm /hand muscles are able to learn writing at normal level for their age.

S50 “Handwriting is essential for 51 learning reading and spelling.” This statement is not true, and also not supported by the reference: yes, it may facilitate reading, but is not a condition to be able to read. Please skip this sentence: S 54 is enough as statement: “ Handwriting may facilitate learning reading and spelling.”

P37 S98-99: “WRITIC-TP, Timed-TIHM 99 and 9-HPT” Why did you change the order of the tests in comparison to the introduction before? You keep this order in the “Instruments” section, so please change the order in the introduction.
