# Supplementary material for: Ready for handwriting? A reference data study on handwriting readiness assessments
Source: PLoS One. 2023 Mar 3;18(3):e0282497. doi: 10.1371/journal.pone.0282497 (PMC9983835; doi:10.1371/journal.pone.0282497)
Supplement: Attachment — Submitted filename: Response to reviewers PONE-D-22-29322R1.docx [file pone.0282497.s005.docx]

Response to reviewers PONE-D-22-29322R1

We would like to thank the editor and the reviewers for their very quick review of our revised manuscript and their positive evaluation. Below, we give a point-by-point response to the remaining concerns and describe how we have addressed these in our revised manuscript. The original remarks are printed in normal font, our response is given in *italics.* Changed parts of the original text are quoted and identified using ‘track changes’ in the revised manuscript.

Comments editor:

1) Ref 3: in summary: “Preliterate, five-year old children printed, typed, or traced letters and shapes, then were shown images of these stimuli while undergoing functional MRI scanning. A previously documented "reading circuit" was recruited during letter perception only after handwriting-not after typing or tracing experience. These findings demonstrate that handwriting is important for the early recruitment in letter processing of brain regions known to underlie successful reading. Handwriting therefore may facilitate reading acquisition in young children.

The reading circuit was only activated by handwriting in healthy children.

However, there are dyslexic children who can read at normal level but are of very low level in writing / spelling. Children with weakness of the arm /hand muscles are able to learn writing at normal level for their age.

S50 “Handwriting is essential for 51 learning reading and spelling.”  This statement is not true and also not supported by the reference: yes, it may facilitate reading, but is not a condition to be able to read. Please skip this sentence: S 54 is enough as statement: “Handwriting may facilitate learning reading and spelling.”

*Reply: Thank you for your comment. We agree that the statement that handwriting facilitates reading and spelling is more adequate and have deleted the sentence in S50.*

2) P37 S98-99: “WRITIC-TP, Timed-TIHM 99 and 9-HPT.” Why did you change the order of the tests in comparison to the introduction before?  You keep this order in the “Instruments” section, so please change the order in the introduction.

*Reply: Thank you for your sharp view. This change was made unintentionally. We agree that it is better to keep the same order as stated in the instrument section. We changed it throughout the whole abstract.*
